# Core Perturbomes of *Escherichia coli* and *Staphylococcus aureus* Using a Machine Learning Approach

**DOI:** 10.3390/pathogens14080788

**Published:** 2025-08-07

**Authors:** José Fabio Campos-Godínez, Mauricio Villegas-Campos, Jose Arturo Molina-Mora

**Affiliations:** Centro de Investigación en Enfermedades Tropicales, Centro de Investigación en Hematología y Trastornos Afines, Facultad de Microbiología, Universidad de Costa Rica, San José 30305, Costa Rica; jose.camposgodinez@ucr.ac.cr (J.F.C.-G.);

**Keywords:** core perturbome, *Escherichia coli*, *Staphylococcus aureus*, machine learning, gene expression, classification, feature selection

## Abstract

The core perturbome is defined as a central response to multiple disturbances, functioning as a complex molecular network to overcome the disruption of homeostasis under stress conditions, thereby promoting tolerance and survival under stress conditions. Based on the biological and clinical relevance of *Escherichia coli* and *Staphylococcus aureus*, we characterized their molecular responses to multiple perturbations. Gene expression data from *E. coli* (8815 target genes—based on a pangenome—across 132 samples) and *S. aureus* (3312 target genes across 156 samples) were used. Accordingly, this study aimed to identify and describe the functionality of the core perturbome of these two prokaryotic models using a machine learning approach. For this purpose, feature selection and classification algorithms (KNN, RF and SVM) were implemented to identify a subset of genes as core molecular signatures, distinguishing control and perturbation conditions. After verifying effective dimensional reduction (with median accuracies of 82.6% and 85.1% for *E. coli* and *S. aureus*, respectively), a model of molecular interactions and functional enrichment analyses was performed to characterize the selected genes. The core perturbome was composed of 55 genes (including nine hubs) for *E. coli* and 46 (eight hubs) for *S. aureus*. Well-defined interactomes were predicted for each model, which are jointly associated with enriched pathways, including energy and macromolecule metabolism, DNA/RNA and protein synthesis and degradation, transcription regulation, virulence factors, and other signaling processes. Taken together, these results may support the identification of potential therapeutic targets and biomarkers of stress responses in future studies.

## 1. Introduction

**Figure 1:**
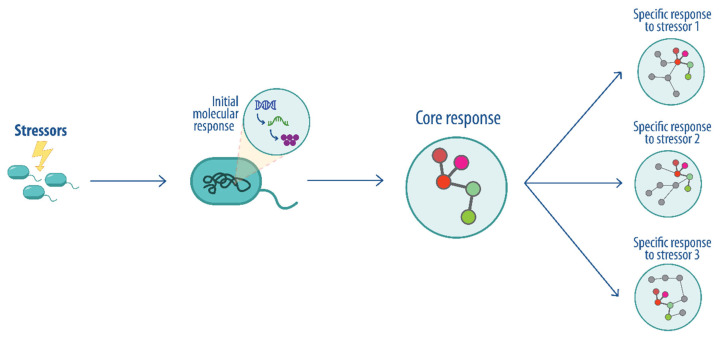
Conceptualization of the core perturbome for biological systems. After exposure to a stressor, general molecular responses (core) are modulated jointly with other specific responses to the perturbation. The gray nodes indicate specific response to a given perturbation.

Biological organisms require complex cellular and molecular interactions to ensure homeostasis and survival [1]. Several studies have revealed diverse molecular mechanisms that are coordinated through interaction networks and can explain the response to disturbances in many organisms [1,2,3,4,5]. Thus, developing new strategies for studying these interactions has been fundamental for understanding the biological processes and identifying different genotypic and phenotypic patterns, defined as consistent molecular changes, that serve as biomarkers of stress or biological states [6]. In this context, and given the importance of understanding central responses to cellular stress across organisms, the concept of the *perturbome* has been coined. Metabolic and signal transduction pathways are modulated following exposure to different perturbations, including a subset of shared or core pathways that are independent of the specific stressor, collectively referred to as the core perturbome (Figure 1), as shown in previous studies [1,7]. In one such study, a human cell model was used to describe diverse stress-response genes active upon drug exposure, suggesting the presence of a central control mechanism. The authors applied a framework to a large-scale imaging screen of cell morphology changes induced by diverse drugs and their combination, resulting in a network of 242 drugs and 1832 interactions [1]. In prokaryotes, the first perturbome was described for *Pseudomonas aeruginosa*, including a machine learning strategy implemented using a benchmarking strategy based on multiple data partition schemas and several classifiers to select genes guided by model performance metrics. The analysis identified 46 genes as part of the central response to perturbations, with biological functions related to biosynthesis, binding, and metabolism, DNA damage repair and aerobic respiration in the context of tolerance to stress [7].

At the transcriptional level, multiple studies have shown that distinct molecular responses can be detected within gene networks that are specific to each perturbation. These responses are closely linked to the modulation of various metabolic pathways, ensuring functional redundancy and robustness in the face of diverse stress stimuli [1,3,7]. Key contributors to stress responses include genes related to the SOS system (*lexA*, *recA*, *dinB*, *umuDC*, etc.) [8,9,10] and the general stress response mediated by RpoS response (sigma factor *rpoS*, *RNAP*, *xthA*, etc.) [8,11,12], whose roles have been extensively documented.

Although studies explicitly using the term “perturbome” are still limited, the concept has been successfully applied in cellular models in both eukaryotes and prokaryotes. In eukaryotic systems, direct relationships between functionally similar drugs and a specific cellular response have been established [1]. Other studies have also shown neuronal responses through molecular networks triggered by defined stressors [3]. In prokaryotes, related investigations have been conducted in *Escherichia coli* [4,5].

On the other hand, therapeutic interventions, such as antibiotics and biocides, are among the most potent stressors acting on bacterial pathogens. These agents disrupt microbial homeostasis and impose strong selective pressures, ultimately threatening the survival of the microorganisms [13].

However, the emergence of pathogens resistant to antibiotics and biocides constitutes a public health concern and is currently among the most significant critical global challenges [14,15]. In this context, elucidating the central molecular response to perturbations offers a valuable opportunity not only to describe the physiological strategies that bacteria employ to survive under stress conditions but also to identify potential biomarkers and therapeutic targets [7].

In this work, we investigated the molecular determinants of the core perturbome in *E. coli* and *Staphylococcus aureus* models. *E. coli* is a gram-negative, facultative anaerobic bacterium commonly found in various environments and involved in various infections across distinct hosts [16,17]. Over several decades, a vast arsenal of resistance genes has been found in *E. coli*, suggesting that this genus serves as a critical reservoir of determinants related to antibiotic resistance [18]. The second model, *S. aureus*, is a ubiquitous, gram-positive, facultative anaerobe frequently implicated in both nosocomial and community-acquired infections in humans and animals [19]. Of particular concern are methicillin-resistant *S. aureus* strains (MRSA), which are associated with high morbidity and mortality rates worldwide [20].

Given the public health relevance of these bacterial models and the increasing availability of high-throughput molecular technologies (e.g., microarrays and massively parallel sequencing), there is a pressing need for innovative computational strategies capable of handling and interpreting large-scale, complex datasets. In this regard, artificial intelligence (specifically machine learning) has emerged as a powerful tool for detecting and describing nontrivial patterns in massive molecular datasets [21]. Several studies have used machine learning algorithms to evaluate the impact of stressors on various biological organisms by identifying specific molecular responses, including models based on feature selection and classification tasks for accurate prediction of cellular states and the discovery of potential biomarkers from transcriptomic data [7]. Thus, machine learning has played a crucial role in uncovering patterns within molecular networks, enabling the identification of key and hub genes, pathways, and interactions that underlie complex biological responses in both eukaryotic organisms [22,23,24] and prokaryotes, such as *Bacillus subtilis* [25] and *Listeria monocytogenes* [26].

Overall, this study proposed to explore the perturbomes of *E. coli* and *S. aureus* through machine learning (specifically feature selection and classification) with transcriptomic data. Gene expression data from *E. coli* (8815 target genes based on pangenome, across 132 samples) and *S. aureus* (3312 target genes across 156 samples) were used. We hypothesized that the bacteria exposed to various perturbations would exhibit distinct transcriptomic signatures but would reveal a core molecular response characterized by the enrichment of metabolic and signal transduction pathways involved in stress responses. Based on this hypothesis, the specific goal was to identify and functionally characterize genes commonly associated with different perturbations in two prokaryotic models using machine learning.

## 2. Materials and Methods

**Figure 2:**
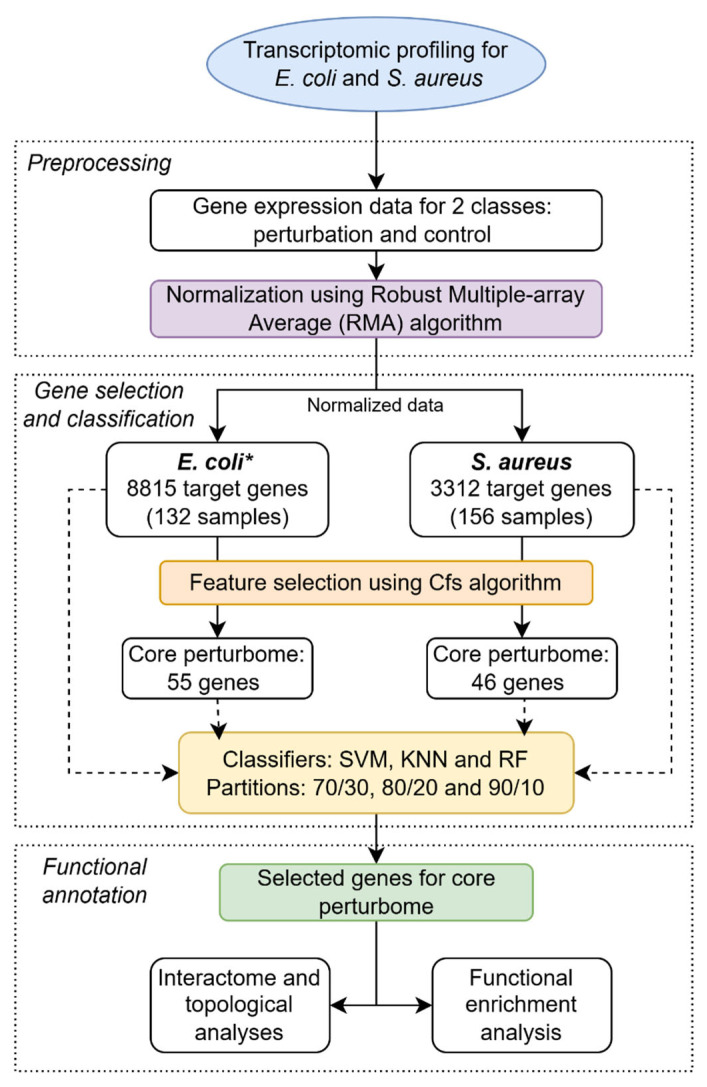
General pipeline for identifying core perturbome in *E. coli* and *S. aureus* by a machine learning approach. * For *E. coli*, microarray was designed based on the pangenome with four strains.

The general strategy followed in this work is presented in Figure 2.

### 2.1. Selection of Biological Models and Transcriptomic Data

Two biological models, *E. coli* and *S. aureus*, were selected for this study. Publicly available transcriptomic datasets were retrieved from the Gene Expression Omnibus (GEO) database (https://www.ncbi.nlm.nih.gov/geo/, accessed on 5 March 2021). For each organism, the high-throughput molecular platforms with the largest number of experiments and samples with available data were chosen:

- *E. coli*—GPL3154: 8815 probes (target genes) after intergenic elements were excluded. Note: Annotated genomes of *E. coli* strains typically report > 4200 genes, but the Affymetrix microarray covers genes of the pangenome of four strains; details at the following website: https://www.ncbi.nlm.nih.gov/geo/query/acc.cgi?acc=GPL3154;
- *S. aureus*—GPL1339: 3312 probes (target genes). Note: microarray based on a single genome; details at the following website: https://www.ncbi.nlm.nih.gov/geo/query/acc.cgi?acc=GPL1339.

The inclusion criteria for experiments and samples in each platform were as follows: (i) having information available regarding the type of perturbation to which the bacteria were exposed (antibiotics, detergents, or chemicals), (ii) having similar culture conditions, and (iii) having control conditions (i.e., unexposed to perturbations).

For *E. coli*, the final gene expression dataset was composed of 9 series with 87 samples for perturbations and 45 controls. For *S. aureus*, the final dataset was composed of 15 series with 92 perturbation cases and 64 controls. Descriptions of the datasets, including accession numbers, types of perturbations, and numbers of cases, are presented in Table 1.

### 2.2. Normalization

Transcriptomic data files (TAR format) were retrieved from the GEO database using Bioconductor (https://www.bioconductor.org/) in R software v4.2.2 (https://r-project.org/) with RStudio v2022.12.0 (https://rstudio.com) using classical functions for microarrays. Background correction, normalization, and summarization were performed with the Robust MultiArray Average algorithm (RMA) with the Affy package in Bioconductor [27].

### 2.3. Machine Learning Algorithms

Machine learning analyses were performed using the Caret package (caret.r-forge.r-project.org/) in RStudio/R software. In the first step, based on the complete dataset for each model, a feature selection approach was used to identify the most relevant genes contributing to each condition (control vs. perturbation). For this purpose, the correlation-based feature selection algorithm (Cfs) was used to reduce dimensionality [28], which identifies the most relevant features (genes) for distinguishing between classes. This approach selects a subset of features that are highly correlated with the target class (perturbations versus controls) but exhibit low intercorrelation among themselves.

In a second analysis, three classification algorithms were used to assess the effectiveness of dimensionality reduction based on the performance of the subset of genes in differentiating between the control and perturbation groups: support vector machine (SVM, kernel = “svmRadial”, epsilon = 0.1, complexity_C = 1.0, tolerance = 0.001) [29], K-nearest neighbors (KNN, algorithm = “LinearNNSearch”, Number_neighbours = 1) [30], and random forest (RF, num_slots = 1, bag% = 100, iterations = 100) [31]. Parameter tuning involved the use of the train() function and the “tuneGrid” option, in which specific model-dependent parameters were selected to be optimized. For RF, mtry (number of variables randomly sampled at each split) was tuned, while k (number of neighbors) was optimized for KNN. For SVM, cost (c) and sigma values were evaluated. Moreover, other classifiers were initially tested (logistic regression, rpart, logit-boost, and neural network) but were excluded after comparison (the three best cases at the training stage were selected for further analysis). All these algorithms considered a 10-fold cross-validation for training, similar to [7]. Due to the dependency of the results on data partitioning, three splits were applied for the training and testing steps: 70/30 (70% training and 30% testing), 80/20, and 90/10. These conditions were applied before and after gene selection. Performance metrics, including accuracy, kappa, precision, recall, true positives (TPs), false positives (FPs), and area under the receiver operating characteristic curve (AUC), were calculated. Selected genes were considered the key elements of the central response to multiple perturbations, i.e., the core perturbome members for each bacterial model.

### 2.4. Molecular Interactions and Functional Enrichment

Based on the list of candidate genes, corresponding identifiers, biological functions, and protein-level sequences were retrieved from the UniProt database (https://www.uniprot.org/id-mapping, accessed on 5 March 2021). Using a systems biology approach, sequences were employed to construct a model of molecular interactions (interactome) with the Search Tool for the Retrieval of Interacting Genes database (STRINGdb, https://string-db.org/) [32]. The interaction models were generated using default settings, incorporating evidence from experiments, co-expression, gene co-occurrence, text mining, and others, as well as a minimum required interaction score of 0.150.

The resulting graph was exported and visualized using Cytoscape software v 3.7.1 [33]. Hub genes were identified with the Cytohubba plugin [34] based on the top 5 nodes with the best values for degree, betweenness, and bottleneck topological metrics.

Finally, to investigate functional enrichment, protein sequences were analyzed using the Kyoto Encyclopedia of Genes and Genomes (KEGG) database and the tool KOALA (KEGG Orthology And Links Annotation, https://www.kegg.jp/blastkoala/, accessed on 5 March 2021, version 3.0) [35], allowing the identification of functional modules among the selected genes.

## 3. Results

### 3.1. Core Perturbome Genes of E. coli and S. aureus Can Be Identified Using a Machine Learning Approach

Transcriptomic microarray data comprising 8815 genes from *E. coli* (based on the pangenome from four strains) and 3312 genes from *S. aureus* (based on a single genome) were preprocessed for machine learning analyses. The Cfs feature selection algorithm was implemented to identify key elements capable of distinguishing between experimental classes (control vs. perturbations). Following this dimensionality reduction, a substantial decrease in the number of genes was achieved: 55 genes (0.62%) for *E. coli* and 46 for *S. aureus* (1.39%).

Three classification algorithms (SVM, KNN, and RF) and three data partitions (70/30, 80/20, and 90/10) were applied before and after dimensionality reduction. The accuracy or percentage of correctly classified instances was determined for each case, as shown in Table 2.

Among all algorithm–partition combinations for *E. coli*, the median accuracy was 56.52% when using the full dataset (8815 genes), but this value drastically increased to 82.61% when using the selected subset of 55 genes. Similarly, for *S. aureus*, the median accuracy was 74.5% for the complete dataset (3312 genes), which increased to 85.1% after feature selection (46 genes). Furthermore, under partitioning variations and algorithms, for both biological models, the best combination was the KNN classifier with a 90/10 partition. Nonetheless, other classifiers also performed satisfactorily after dimensionality reduction, with most configurations achieving an accuracy of above 70%. More details are provided in Table 2.

Given that model performance should not rely solely on accuracy, additional evaluation metrics were used to compare the classification models after dimensionality reduction, as shown in Table 3. Depending on the metric, RF outperformed other classifiers in several cases. For instance, RF yielded superior values for the kappa value, TP rate, F score, and AUC across multiple partitions for both biological models. Again, the results for other conditions (partitions and algorithms) also showed acceptable performance after gene selection, reinforcing the robustness of the selected features and classification strategy.

### 3.2. Biological Functions and Well-Defined Interactions Can Be Recognized for Genes of the Core Perturbome

Following gene selection, biological functions of the corresponding proteins were identified for each candidate gene, as shown in Table 4 and Table 5. Genes related to metabolic processes, transport, transcriptional regulators, and virulence factors were found in each model. More details are presented in the Supplementary Material. Subsequently, a systems biology approach was employed to construct molecular interaction networks for each model.

**Figure 3:**
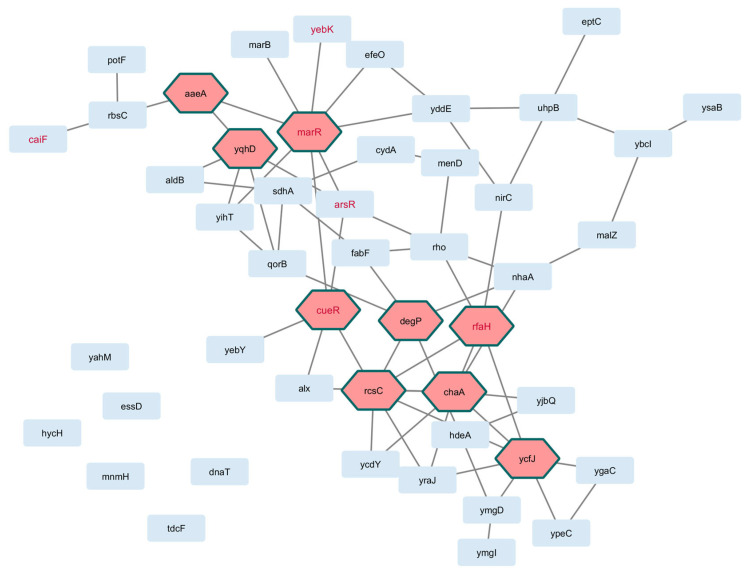
Interactome of genes in the core perturbome of *E. coli.* Annotation of each gene product (light blue nodes) was used to model molecular interactions, resulting in 42 connected elements with 9 hub genes (pink nodes) as key determinants of the network. Red-label nodes identify transcription factors.

**Figure 4:**
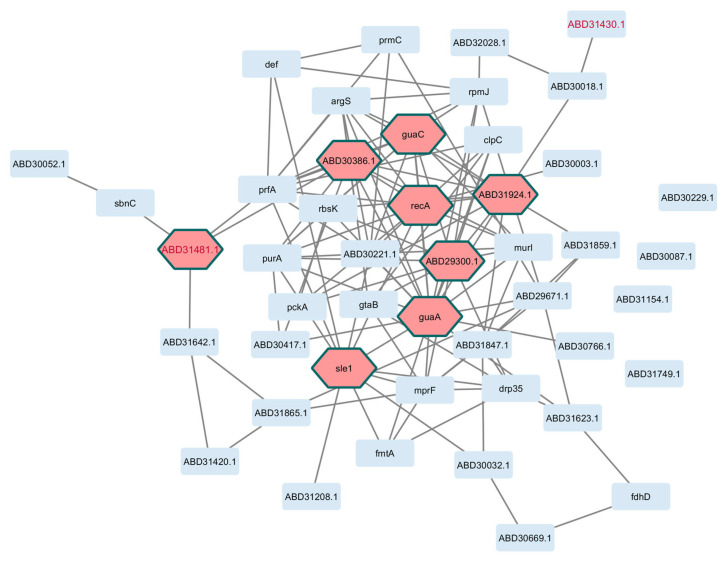
Interactome based on genes of the core perturbome in *S. aureus.* Annotation of each gene product (light blue nodes) was used to model molecular interactions, resulting in 42 connected elements with 8 hub genes (pink nodes) as key determinants of the network. Red-label nodes identify transcription factors.

As shown in Figure 3 and Figure 4 for *E. coli* and *S. aureus*, respectively, well-defined interactions were obtained. For *E. coli*, topological metrics indicated that 42 nodes out of 55 selected genes (76.4%) were connected with 67 edges in total (six nodes were not connected, and six were not mapped to the database); nine hub gene products were recognized in the network, namely, marR, cueR, ecsC, and ycfJ. For *S. aureus*, 42 nodes out of 46 genes (91.3%) were connected through 123 edges (four unconnected genes), and the gene products recA, guaA, and sleA were among the eight hub genes in this interactome.

**Figure 5:**
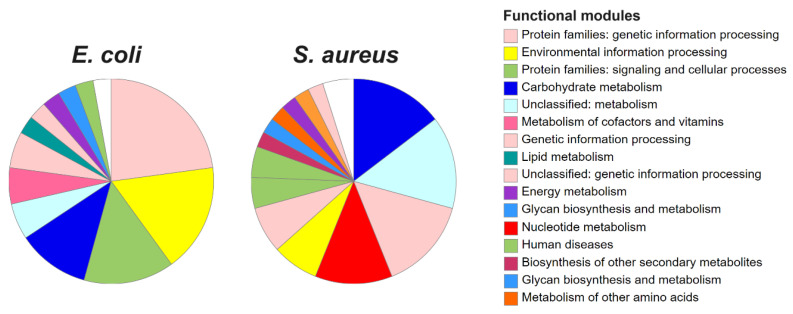
Functional enrichment of genes in the core perturbome of *E. coli* and *S. aureus.* Annotation was based on KEGG ontologies, indicating modulation pathways related to metabolism, protein synthesis, transcription factors and others.

Finally, functional enrichment analysis based on KEGG ontologies revealed a diverse array of biological pathways shared across both models. These included energy and macromolecule metabolism, DNA/RNA and protein synthesis and degradation, transcription regulation, virulence factors, and pathways associated with human diseases (pathogenesis), and others, as depicted in Figure 5 and Table 6. These results were based on 35 (63.6%) annotated genes for *E. coli* and 41 (89.1%) annotated entries for *S. aureus*. Despite differences in the identities of the selected genes in the core perturbome in each model, both bacteria exhibited similar patterns of enriched biological modules (Table 6), suggesting conserved strategies in the bacterial response to stress.

## 4. Discussion

The core perturbome is defined as the central molecular response of an organism to multiple external disturbances [1,7]. This response functions as a complex molecular network that counteracts disruptions in cellular homeostasis, thereby promoting tolerance and survival under stress conditions [36]. In this study, we focused on the core perturbomes of *E. coli* and *S. aureus*, two bacterial species frequently implicated in infections across several hosts, including critical cases in humans with multidrug-resistant strains [4,37,38]. Characterizing the molecular responses to perturbations in those pathogens can provide valuable insights into potential therapeutic targets and biomarkers [39].

As in other studies, transcriptomic data served as a powerful resource for determining key elements involved in stress response, based on changes in gene expression and associations with phenotypic outcomes [40,41,42]. Although gene expression patterns have been studied via machine learning in other biological contexts [24,43,44,45,46], reports on the central response to stimuli in prokaryotic models remain limited. Some reports exist for *Bacillus subtilis* [25] and *Listeria monocytogenes* [26], while systematic analyses under the core perturbome framework for *E. coli* and *S. aureus* are largely absent. Alternative approaches have explored responses to multiple stressors in *E. coli* [5] and *S. aureus* [47], but comprehensive perturbome-level investigations have yet to be reported.

In our analysis, machine learning was used to identify core molecular signatures distinguishing control and perturbation conditions. Feature selection was performed using the Cfs algorithm, which removed irrelevant and redundant features, thereby enhancing the classifiers’ performance. This approach yielded a reduced set of genes with high predictive power: 55 (with nine hubs) for *E. coli* and 46 (with eight hubs) for *S. aureus*. These results are in line with previous reports in terms of magnitude. For example, network analysis revealed 24 central genes in *E. coli* [5], 122 genes in the sigmaB regulon of *S. aureus* [48], and 46 genes in the perturbome of *Pseudomonas aeruginosa* [7].

The assessment of selected genes using classification algorithms (SVM, RF, and KNN) and data partitions (70/30, 80/20, and 90/10) demonstrated a substantial improvement in model performance after feature selection in both prokaryotic models. The median accuracy was 82.6% for *E. coli* and 85.1% for *S. aureus* after dimensional reduction, in contrast to the median accuracies of 56.52% and 74.5%, respectively, for complete datasets. These results suggest that the selected subset of genes not only retained sufficient discriminatory power to classify the samples accurately but reduced noise effectively—an expected outcome of successful dimensional reduction. In transcriptomic profiling using massive amounts of molecular data, the extraction of relevant information and reducing noise by selecting a subset of relevant genes are still open problems [49]. Our approach, combining feature selection with robust machine learning classifiers, effectively addressed this challenge. The use of SVM, RF, and KNN, which usually outperform other classifiers in comparative strategies [50,51,52], was key to this success. In contrast, the other four classifiers (logistic regression, rpart, logit-boost, and neural network) that demonstrated suboptimal performance were excluded early in the analysis. For the selected classifiers, performance showed some differences across bacterial species. Based on all the metrics, and despite not large differences among classifiers, RF outperformed other algorithms for *E. coli*, while SVM showed superior performance for *S. aureus*. This situation is a common and expected behavior for different datasets (from two very different models, distinct microarray platforms, and diverse wet lab experiments used to generate transcriptomic data), as previously reported [30,46,53]. Given the data variability introduced by processing assays across different laboratories (different GEO projects), traditional approaches like differential expression analysis were unsuitable. A more robust method was, therefore, required to account for this variability, and machine learning proved effective in identifying meaningful patterns under these conditions.

Regarding the biological functionality of the selected genes, an orchestrated response was observed to work synergistically based on the modulation of metabolic pathways with interrelated genes, supported by gene annotation, network analysis, and functional enrichment analyses. In the case of hub genes, these are decisive regulators for transferring regulatory information through signaling, functioning as activators or repressors of case-specific operons/genes. Hubs not only have many connections with other genes within the network but also influence the expression and function of many other genes, acting as control centers in the network. Notably, transcription factors played a central role: six in *E. coli* (ybbI -hub-, caiF, RfaH -hub-, HexR, arsR, and marR -hub) and two in *S. aureus* (mtlR/SACOL2147 and MerR/SACOL2193 -hub-). The regulatory functions of these genes are associated with the control of efflux pump activity, porin expression, DNA repair mechanisms, and macromolecule transport, which together counteract the effects of antibiotics and genotoxic agents [54,55,56]. These regulated elements were found in our analysis. Furthermore, genes linked to protein synthesis, modulation of growth, and virulence factors were also identified in both bacterial models. These biological functions are consistent with other studies, indicating that these routes are involved in the physiological and metabolic changes that contribute to the tolerance, resisting stress and ensuring only essential functions to survive under stressful conditions [56,57].

Although no specific genes are directly linked to classical stress responses, such as the SOS or RpoS responses, several key pathways functionally related to these responses were enriched in the core perturbomes of *E. coli* and *S. aureus* [5,58,59]. For example, functional and enriched pathways associated with “DNA damage repair”, “energy and macromolecule metabolism”, “DNA/RNA and protein synthesis and degradation”, “transcription regulation”, “virulence factors”, and other signaling processes were consistently enriched in both models. These biological modules are well-documented components of bacterial adaptation under stress conditions [4,54,55,60]. Although part of the transcriptomic data is from non-pathogenic strains (*E. coli* K12, for example, as well as the consideration of genes from the pangenome with four strains), the selected genes largely belong to the conserved core genome, implying that these determinants likely play similar roles in pathogenic lineages.

Interestingly, the number of enriched pathways was relatively limited, which may reflect redundancy and robustness in the general response to stress [4,7]. This has been reported in other works. For instance, our previous study of the core perturbome of *P. aeruginosa* revealed 46 genes and a reduced number of pathways associated with biosynthesis, protein binding, and metabolism, many of which are related to DNA damage repair and aerobic respiration in the context of tolerance to stress [7]. In the study by [5] with *E. coli*, interactome analysis of 24 central proteins revealed the role of RNA binding, virulence factors, transporters, and DNA repair as important processes during the stress response.

Furthermore, the biological significance of perturbome analysis was underscored in our prior research with *P. aeruginosa* strain AG1 [61]. There, we identified core genes and those exclusively induced by ciprofloxacin exposure. Most of the genes that were not part of the core perturbome were resident prophages of the genome. These determinants were expressed in response to ciprofloxacin but not to other antibiotics. This observation, validated phenotypically, supports the potential utility of phages as endogenous modulators in therapeutic strategies, including phage therapy [61].

Moreover, a preliminary ortholog comparison among the studied models (*P. aeruginosa* from the previous study and *E. coli* and *S. aureus* from the present work) using OrthoFinder [62] revealed six shared genes (*rho*, *fabF*, *tdcF*, *argS*, *sle1*, and *gtaB*), suggesting a conserved role in the molecular stress response across these species. This is considered a robust selection from different bacteria and diverse experimental conditions to obtain transcriptomic data within each bacterial species. We are currently studying them by molecular docking to evaluate their druggability and eventually predict the in silico and in vitro effects using chemical compounds on the modulation of stress tolerance. These genes are being structurally modeled using PDB or AlphaFold, and in collaboration with a structural biology team, we are working to identify chemical inhibitors. We are also standardizing PCR protocols to quantify gene expression of the core perturbome for *E. coli* and *S. aureus*, as it was established for *P. aeruginosa* recently [63].

Regarding the limitations, this study was primarily affected by data availability, in which specific platforms were used to obtain complete data with comparable information. For example, for *E. coli*, the microarray data used were from a non-pathogenic strain (K-12 substrain MG1655) and the microarray platform was designed based on the pangenome with four strains; selection was based on data availability rather than relevance as a pathogen or obtained with more recent and advanced technologies such as RNA sequencing. Future analyses would benefit from incorporating RNA-seq data from pathogenic strains, ideally obtained using the same platform and under comparable experimental conditions to enhance consistency and biological relevance. Furthermore, the included transcriptomic datasets were generated under heterogeneous experimental setups, which may introduce variability. Although strict inclusion criteria were applied to minimize this source of bias, the lack of uniform conditions remains a limitation. Finally, while this study provides insights into gene expression responses at the transcriptomic level, additional validation of the identified genes through proteomic analyses and comprehensive phenotypic assays is necessary to confirm their functional roles in bacterial stress responses.

## 5. Conclusions

In conclusion, this study identified the core perturbomes of *E. coli* and *S. aureus* through a machine learning-based approach with transcriptomic data. Feature selection enabled effective dimensionality reduction, improving classification performance to median accuracies of 82.6% and 85.1% for *E. coli* and *S. aureus*, respectively, across multiple data partitions (70/30, 80/20, and 90/10). The core perturbomes comprised 55 genes (including nine hubs) for *E. coli* and 46 genes (including eight hubs) for *S. aureus*, including both old-acquaintance regulators (such as transcription factors) and new possible determinants of the response to stress. Functional and network analyses revealed enrichment in key biological processes, including pathways related to energy and macromolecule metabolism, DNA/RNA and protein synthesis and degradation, transcription regulation, virulence, and other signaling processes. These results provide new insights into the conserved and strain-specific molecular mechanisms that underpin bacterial adaptation to diverse stressors, offering a foundation for future research on antimicrobial targets and stress resilience in prokaryotes.

## Figures and Tables

**Table 1:** Description of datasets used to study the perturbomes of *E. coli* and *S. aureus* (from the NCBI-GEO platform).

Model	GEO-ID	Perturbation	Strain	Number of Samples	
				Control	Perturbation
*E. coli*	GSE10159	Cefzulodin, mecillinam	K12 MG1655	20	41
	GSE10160				
	GSE10345	Bicyclomycin	K12 MG1655	2	6
	GSE13982	Carbon monoxide	K12 MG1655	4	4
	GSE34275	Glycerol	K12 MG1655	6	6
	GSE37026	Colicine	K12 MG1655	4	4
	GSE44211	PGRP, gentamicin, CCCP	K12 MG1655	3	9
	GSE53140	Octanoic acid	K12 MG1655	3	2
	GSE56133	Ampicillin, gentamicin, kanamycin, norfloxacin, H_2_O_2_	K12 MG1655	3	15
*S. aureus*	GSE7944	Berberine chloride	ATCC25923	3	3
	GSE8135	Rhein/cassic acid	ATCC25923	3	3
	GSE8861	Triclosan	NCTC8325 WT	5	10
	GSE10605	Ortho-phenylphenol	NCTC8325 WT	5	10
	GSE13203	Cryptotanshinone	ATCC25923	3	3
	GSE13233	Sodium houttuyfonate	ATCC25923	3	3
	GSE13236	Magnolol	ATCC25923	3	3
	GSE14669	Ramoplanin	NCTC 8325	6	6
	GSE15394	Fosfomycin	ATCC 29213	14	23
	GSE36231	Oleic acid	NCTC8325	3	3
	GSE40448	Ortho-Benzyl-Para-Chloro Phenol	NCTC 8325	5	10
	GSE40449	Para-Tert-Amylphenol	NCTC 8325	4	8
	GSE58938	Licochalcone A	ATCC 29213	2	2
	GSE65750	Nisin	ATCC 29213	2	2
	GSE84485	Benzimidazole derivative C162	NCTC8325, ATCC25923	3	3

**Table 2:** Assessment of the performance of the classification models before and after dimensionality reduction of the transcriptomic data for *E. coli* and *S. aureus*.

Model	Partition	Gene Dataset (Number of Genes)	Correctly Classified Instances (%)		
			KNN	SVM	RF
*E. coli*	70/30	All (8815)	67.7	67.7	44.1
		Selected genes (55)	82.5	70.6	88.2
	80/20	All (8815)	56.5	65.2	39.1
		Selected genes (55)	82.6	91.3	91.3
	90/10	All (8815)	54.6	81.8	54.6
		Selected genes (55)	90.9	81.8	81.8
*S. aureus*	70/30	All (3312)	74.5	63.8	80.9
		Selected genes (46)	85.1	91.5	78.7
	80/20	All (3312)	61.3	74.2	74.2
		Selected genes (46)	77.4	80.6	74.2
	90/10	All (3312)	87.5	87.5	100.0
		Selected genes (46)	93.8	93.7	87.5

**Table 3:** Assessment of the performance of the classification models after dimensionality reduction of the transcriptomic data for *E. coli* and *S. aureus* using different metrics.

Model	Metrics	KNN			SVM			RF		
		70/30	80/20	90/10	70/30	80/20	90/10	70/30	80/20	90/10
*E. coli*	Accuracy	82.5	82.6	90.9	70.6	91.3	81.8	88.2	91.3	81.8
	Kappa	65.0	65.4	81.3	42.9	82.7	62.0	76.5	82.5	87.1
	TP rate	82.4	82.6	90.9	70.6	91.3	81.8	88.2	91.3	93.8
	FP rate	16.4	16.7	10.9	26.1	8.0	21.8	11.2	8.7	3.8
	Precision	84.5	83.7	92.2	81.9	92.6	86.4	88.8	91.3	94.6
	Recall	82.4	82.6	90.9	70.6	91.3	81.8	88.2	91.3	93.8
	F score	82.2	82.5	90.8	68.4	91.3	80.8	88.2	91.3	93.8
	AUC	83.0	83.0	90.0	72.2	91.7	80.0	96.9	98.1	100.0
*S. aureus*	Accuracy	85.1	77.4	93.8	91.5	80.6	93.7	78.7	74.2	87.5
	Kappa	70.0	55.0	87.1	82.4	58.4	87.1	55.3	44.6	73.3
	TP Rate	85.1	77.4	93.8	91.5	80.6	93.8	78.7	74.2	87.5
	FP Rate	14.8	20.6	3.8	10.5	24.7	3.8	25.4	31.5	14.2
	Precision	85.2	79.2	94.6	92.6	82.1	94.6	81.9	74.8	87.5
	Recall	85.1	77.4	93.8	91.5	80.6	93.8	78.7	74.2	87.5
	F score	85.1	77.6	93.8	91.3	79.9	93.8	77.6	73.1	87.5
	AUC	85.2	78.4	95.0	99.1	96.2	100.0	76.6	71.4	86.7

**Table 4:** Gene annotation for elements of the core perturbome in *E. coli*.

ID (Array)	Gene Names	Protein ID	StringID	Annotation
c0820	c0820	A0A0H2V608	Not mapped	Uncharacterized protein
c1618	c1618	A0A0H2V6W5	199310.c1618	YmgI protein
c1419	c1419	A0A0H2V7D7	Not mapped	Uncharacterized protein
c2755	c2755	A0A0H2V917	Not mapped	Uncharacterized protein
c4081	c4081	A0A0H2VBA2	199310.c4081	Uncharacterized protein
c4088	c4088	A0A0H2VBF5	Not mapped	Uncharacterized protein
c4086	c4086	A0A0H2VE07	Not mapped	Uncharacterized protein
uhpB	uhpB b3668 JW3643	P09835	511145.b3668	Sensor histidine protein kinase UhpB
dnaT	dnaT b4362 JW4326	P0A8J2	511145.b4362	Primosomal protein DnaT
ybbI (hub)	cueR copR ybbI b0487 JW0476	P0A9G4	511145.b0487	Transcriptional regulator cueR, transcription factor
c1561	essD ybcR b0554 JW0543	P0A9R2	511145.b0554	Lysis protein S homolog from lambdoid prophage DLP12
fabF	fabF fabJ b1095 JW1081	P0AAI5	511145.b1095	3-oxoacyl-[acyl-carrier-protein] synthase II
ycdO	efeO ycdO b1018 JW1003	P0AB24	511145.b1018	Iron uptake system component EfeO
ycfJ (hub)	ycfJ b1110 JW1096	P0AB35	511145.b1110	Hypothetical protein
b1171	ymgD b1171 JW5177	P0AB46	511145.b1171	Hypothetical protein ymgD precursor
cydA	cydA cyd-1 b0733 JW0722	P0ABJ9	511145.b0733	Cytochrome d terminal oxidase polypeptide subunit I
nirC	nirC b3367 JW3330	P0AC26	511145.b3367	Nitrite reductase activity
sdhA	sdhA b0723 JW0713	P0AC41	511145.b0723	Succinate dehydrogenase flavoprotein subunit
ygaC	ygaC b2671 JW2646	P0AD53	511145.b2671	Hypothetical protein
caiF	caiF b0034 JW0033	P0AE58	511145.b0034	Transcriptional regulator of cai operon, transcription factor
hdeA	hdeA yhhC yhiB b3510 JW3478	P0AES9	511145.b3510	Acid stress chaperone HdeA (10K-S protein)
hycH	hycH hevH b2718 JW2688	P0AEV7	511145.b2718	Formate hydrogenlyase maturation protein
yjbQ	yjbQ b4056 JW4017	P0AF48	511145.b4056	Hypothetical protein
rfaH (hub)	rfaH hlyT sfrB b3842 JW3818	P0AFW0	511145.b3842	Transcriptional activator RfaH, transcription factor
rho	rho nitA psuA rnsC sbaA tsu b3783 JW3756	P0AG30	511145.b3783	Transcription termination factor Rho
rbsC	rbsC b3750 JW3729	P0AGI1	511145.b3750	D-ribose high-affinity transport system permease protein
b3113	tdcF yhaR b3113 JW5521	P0AGL2	511145.b3113	Putative reactive intermediate deaminase TdcF
b0161 (hub)	degP htrA ptd b0161 JW0157	P0C0V0	511145.b0161	Serine endoprotease (protease Do), membrane-associated
yijP	eptC cptA yijP b3955 JW3927	P0CB39	511145.b3955	Membrane protein
rcsC (hub)	rcsC b2218 JW5917/JW5920	P0DMC5	511145.b2218	Sensor for ctr capsule biosynthesis
nhaA	nhaA ant b0019 JW0018	P13738	511145.b0019	Na+/H antiporter
menD	menD b2264 JW5374	P17109	511145.b2264	2-oxoglutarate decarboxylase
malZ	malZ b0403 JW0393	P21517	511145.b0403	Maltodextrin glucosidase
marR (hub)	marR cfxB inaR soxQ b1530 JW5248	P27245	511145.b1530	Repressor of mar operon, transcription factor
marB	marB b1532 JW1525	P31121	511145.b1532	Multiple antibiotic resistance protein
potF	potF b0854 JW0838	P31133	511145.b0854	Periplasmic putrescine-binding permease protein
chaA (hub)	chaA b1216 JW1207	P31801	511145.b1216	Sodium-calcium/proton antiporter
yihT	yihT b3881 JW3852	P32141	511145.b3881	Putative aldolase
ybbB	selU ybbB b0503 JW0491	P33667	511145.b0503	Putative capsule anchoring protein
arsR	arsR arsE b3501 JW3468	P37309	511145.b3501	Arsenical resistance operon repressor, transcription factor
aldB	aldB yiaX b3588 JW3561	P37685	511145.b3588	Aldehyde dehydrogenase B
yddE	yddE b1464 JW1459	P37757	511145.b1464	Hypothetical protein
ytfG	qorB qor2 ytfG b4211 JW4169	P39315	511145.b4211	Putative oxidoreductase
yjiT	yjiT b4342 JW5787	P39391	Not mapped	Hypothetical protein
ygjT	alx ygjT b3088 JW5515	P42601	511145.b3088	Putative membrane-bound redox modulator Alx
yraJ	yraJ b3144 JW3113	P42915	511145.b3144	Outer membrane usher protein YraJ
ybcI	ybcI b0527 JW0516	P45570	511145.b0527	Inner membrane protein YbcI
yebK	hexR yebK b1853 JW1842	P46118	511145.b1853	HTH-type transcriptional regulator HexR (Hex regulon repressor), transcription factor
yhcQ (hub)	aaeA yhcQ b3241 JW3210	P46482	511145.b3241	p-hydroxybenzoic acid efflux pump subunit AaeA (pHBA efflux pump protein A)
b1839	yebY b1839 JW1828	P64506	511145.b1839	Uncharacterized protein
c2390	ypeC b2390 JW2387	P64542	511145.b2390	Uncharacterized protein
yahM	yahM b0327 JW5044	P75692	511145.b0327	Uncharacterized protein
ycdY	ycdY b1035 JW1018	P75915	511145.b1035	Chaperone protein YcdY
Z4985	ysaB b4553 JW3532	Q2M7M3	511145.b4553	Uncharacterized lipoprotein YsaB
yqhD (hub)	yqhD b3011 JW2978	Q46856	511145.b3011	Alcohol dehydrogenase YqhD

**Table 5:** Gene annotation for elements of the core perturbome in *S. aureus*.

ID (Array)	Gene Names	Protein ID	StringID	Annotation
SACOL0995	ABD30052.1 SACOL0995	A0A0H2WVP6	93061.SAOUHSC_00927	Oligopeptide ABC transporter, oligopeptide-binding protein
SACOL1539	ABD30669.1 SACOL1539	A0A0H2WW35	93061.SAOUHSC_01590	Cytosolic protein
SACOL1360	ABD30417.1 SACOL1360	A0A0H2WW94	93061.SAOUHSC_01319	Aspartokinase
SACOL1169	ABD30229.1 SACOL1169	A0A0H2WWH1	93061.SAOUHSC_01115	Staphylococcal complement inhibitor
SACOL2193 (hub)	ABD31481.1 SACOL2193	A0A0H2WWP9	93061.SAOUHSC_02461	Transcriptional regulator, MerR family, transcription factor
SACOL1033	ABD30087.1 SACOL1033	A0A0H2WWU8	93061.SAOUHSC_00962	IDEAL domain-containing protein
tcaB	ABD31642.1 tcaB SACOL2350	A0A0H2WX36	93061.SAOUHSC_02633	Bcr/CflA family efflux transporter
SACOL2561	ABD31859.1 SACOL2561	A0A0H2WX88	93061.SAOUHSC_02860	Hydroxymethylglutaryl-CoA synthase
SACOL2731	ABD32028.1 SACOL2731	A0A0H2WXD2	93061.SAOUHSC_03045	Cold shock protein CspA
SACOL2330	ABD31623.1 SACOL2330	A0A0H2WXH1	93061.SAOUHSC_02613	MOSC domain-containing protein
cap5F (hub)	ABD29300.1 cap5F SACOL0141	A0A0H2WXH2	93061.SAOUHSC_00119	Capsular polysaccharide biosynthesis protein Cap5F
SACOL0587	ABD29671.1 SACOL0587	A0A0H2WXZ9	93061.SAOUHSC_00523	Methyltransferase small domain-containing protein
SACOL2551	ABD31847.1 SACOL2551	A0A0H2WY92	93061.SAOUHSC_02846	Acyl-CoA thioesterase
SACOL0959	ABD30018.1 SACOL0959	A0A0H2WYF8	93061.SAOUHSC_00893	NADH-dependent flavin oxidoreductase, Oye family
SACOL2138	ABD31420.1 SACOL2138	A0A0H2WZ64	93061.SAOUHSC_02389	Cation efflux family protein
SACOL2147	ABD31430.1 SACOL2147	A0A0H2WZ69	93061.SAOUHSC_02401	Transcriptional antiterminator, BglG family/DNA-binding protein, transcription factor
SACOL1645	ABD30766.1 SACOL1645	A0A0H2WZH6	93061.SAOUHSC_01692	ComE operon protein 2
SACOL2624 (hub)	ABD31924.1 SACOL2624	A0A0H2WZI5	93061.SAOUHSC_02929	Putative long-chain fatty acid-CoA ligase VraA
SACOL2452	ABD31749.1 SACOL2452	A0A0H2X000	93061.SAOUHSC_02743	Amino acid ABC transporter, permease protein
SACOL2566	ABD31865.1 SACOL2566	A0A0H2X034	93061.SAOUHSC_02866	MmpL efflux pump, putative
SACOL1948	ABD31154.1 SACOL1948	A0A0H2X044	93061.SAOUHSC_02104	Uncharacterized protein
prmC	prmC SACOL2109	A0A0H2X056	93061.SAOUHSC_02358	Release factor glutamine methyltransferase PrmC
SACOL0102	sbnC SACOL0102	A0A0H2X061	93061.SAOUHSC_00077	Siderophore biosynthesis protein, IucC family
clpC	clpC SA0483	Q7A797	93061.SAOUHSC_00505	ATP-dependent Clp protease ATP-binding subunit ClpC
def	def def1 pdf1 SAV1091	P68825	93061.SAOUHSC_01038	Peptide deformylase
drp35	drp35 SACOL2712	Q5HCK9	93061.SAOUHSC_03023	Lactonase drp35
fdhD	fdhD narQ SAV2280	P64120	93061.SAOUHSC_02550	Sulfur carrier protein FdhD
fmtA	fmtA fmt SACOL1066	Q5HH27	93061.SAOUHSC_00998	Teichoic acid D-alanine hydrolase
glnA (hub)	ABD30386.1 glnA SAV1310	P60890	93061.SAOUHSC_01287	Glutamine synthetase
gtaB	gtaB galU SACOL2508	Q5HD54	93061.SAOUHSC_02801	UTP--glucose-1-phosphate uridylyltransferase
guaA (hub)	guaA SAV0391	P64296	93061.SAOUHSC_00375	GMP synthase [glutamine-hydrolyzing]
guaC (hub)	guaC SAV1337	P60562	93061.SAOUHSC_01330	GMP reductase
SAV1152	ABD30221.1 SAV1152	P64309	93061.SAOUHSC_01107	dITP/XTP pyrophosphatase
mprF	mprF SACOL1396	Q5HG59	93061.SAOUHSC_01359	Phosphatidylglycerol lysyltransferase
murI	murI SAV1151	P63637	93061.SAOUHSC_01106	Glutamate racemase
SACOL0944	ABD30003.1 SACOL0944	Q5HHE4	93061.SAOUHSC_00878	Type II NADH:quinone oxidoreductase
SACOL2002	ABD31208.1 SACOL2002	Q5HEI2	93061.SAOUHSC_02161	Membrane protein
pckA	pckA SAV1791	P0A0B3	93061.SAOUHSC_01910	Phosphoenolpyruvate carboxykinase
purA	purA SAV0017	P65884	93061.SAOUHSC_00019	Adenylosuccinate synthetase
rbsK	rbsK SACOL0253	A0A0H2WZY4	93061.SAOUHSC_00239	Ribokinase
recA (hub)	recA SAV1285	P68843	93061.SAOUHSC_01262	Protein RecA
prfA	prfA SAV2118	P66018	93061.SAOUHSC_02359	Peptide chain release factor 1
rpmJ	rpmJ SAV2227	P66298	93061.SAOUHSC_02488	Large ribosomal subunit protein bL36
sle1 (hub)	sle1 aaa SACOL0507	Q5HIL2	93061.SAOUHSC_00427	N-acetylmuramoyl-L-alanine amidase sle1
argS	argS SACOL0663	Q5HI60	93061.SAOUHSC_00611	Arginine--tRNA ligase
SACOL0974	ABD30032.1 SACOL0974	Q5HHB5	93061.SAOUHSC_00907	UPF0344 protein SACOL0974

**Table 6:** Functional enrichment of genes in the core perturbome of *E. coli* and *S. aureus*.

*E. coli*	*S. aureus*
Orthologs and modules ko00001 KEGG Orthology (KO) (35) Protein families: metabolism; ko01000 Enzymes (12); ko01001 Protein kinases (2); ko01002 Peptidases and inhibitors (1); ko01005 Lipopolysaccharide biosynthesis proteins (1); ko01004 Lipid biosynthesis proteins (1). Protein families: genetic information processing ko03000 Transcription factors (6); ko03021 Transcription machinery (1); ko03019 Messenger RNA biogenesis (1); ko03016 Transfer RNA biogenesis (1); ko03110 Chaperones and folding catalysts (2); ko03400 DNA repair and recombination proteins (1). Protein families: signaling and cellular processes ko02000 Transporters (8); ko02044 Secretion system (1); ko02022 Two-component system (2); ko02035 Bacterial motility proteins (1); ko01504 Antimicrobial resistance genes (1).	Orthologs and modules ko00001 KEGG Orthology (KO) (41). Protein families: metabolism ko01000 Enzymes (23); ko01002 Peptidases and inhibitors (1); ko01011 Peptidoglycan biosynthesis and degradation proteins (1); ko01004 Lipid biosynthesis proteins (1); ko01007 Amino acid-related enzymes (1). Protein families: genetic information processing ko03000 Transcription factors (2); ko03011 Ribosome (1); ko03009 Ribosome biogenesis (1); ko03016 Transfer RNA biogenesis (1); ko03012 Translation factors (2); ko03110 Chaperones and folding catalysts (1); ko03400 DNA repair and recombination proteins (1); ko03029 Mitochondrial biogenesis (1). Protein families: signaling and cellular processes ko02000 Transporters (4); ko02044 Secretion system (1); ko04147 Exosome (1); ko01504 Antimicrobial resistance genes (1).

## Data Availability

Public raw data used in this study can be retrieved from the GEO database (https://www.ncbi.nlm.nih.gov/geo/, accessed on 5 March 2025) based on the Series ID reported in Table 1. Pipelines to access data, normalization analysis, machine learning methods, and normalized data are available at: https://github.com/josemolina6/Perturbome.

## Supplementary Materials

The following supporting information can be downloaded at: https://www.mdpi.com/article/10.3390/pathogens14080788/s1, Tables S1 and S2: Gene annotation for elements of the core perturbome; Tables S3 and S4: Correlation of genes and class (control or perturbation); Tables S5 and S6: Gene annotations for the complete dataset in the microarray.

## Abbreviations

The following abbreviations are used in this manuscript:

AUC	Area under the receiver operating characteristic curve
Cfs	Correlation-based feature selection
KNN	K-nearest neighbors
RF	Random forest
SVM	Support vector machine
